# Global Panoramic analysis of clinical research in cell therapy: clinical trial landscape, marketed products, and regulatory trends

**DOI:** 10.3389/fphar.2026.1715984

**Published:** 2026-02-09

**Authors:** Mengmeng Wang, Tiange Zhou, Sijia Liu, Wanwan Xiang, Kewen Xie, Xiaoqiong Zhang, Wenxin Hu, Mengling Fang, Ziyue Zhang, Meimei Chen, Xi Wang, Jiancai Wu

**Affiliations:** 1 Institution for Drug Clinical Trials, Union Hospital, Tongji Medical College, Huazhong University of Science and Technology, Wuhan, China; 2 Fu Foundation School of Engineering and Applied Science, Columbia University, New York, NY, United States

**Keywords:** CAR-T, cell therapy, clinical trials, marketed products, regulatory trends

## Abstract

**Introduction:**

Cell therapy is a vital field in modern medicine. This review assesses its global clinical development landscape, approved products, and regional regulatory characteristics.

**Methods:**

A comprehensive search and analysis of global clinical trial databases (e.g., https://clinicaltrials.gov/ClinicalTrials.gov), regulatory agency announcements, and relevant literature up to October 2025 was conducted. Key data points regarding trial numbers, phases, therapy types, and approved products were extracted and analyzed descriptively by region.

**Results:**

A cumulative total of 10,373 cell therapy clinical trials were identified worldwide, with primary distribution across the United States (3,563 trials), China (3,365 trials), and Europe (1,584 trials). Oncology (56.1%) and immune system diseases (9.3%) were the main research focuses. Immune cell therapy (5,167 trials) and stem cell therapy (4,796 trials) received comparable attention, with CAR-T therapy (2,409 trials) being prominent within the former, and mesenchymal stem cells (MSCs, 1,904 trials) and hematopoietic stem cells (HSCs, 1,550 trials) dominating the latter. Since 2016, China has led in the number of clinical trials, particularly in CAR-T research. Europe had a significantly higher proportion of Phase III trials compared to China and the US. The US led in the approval of HSC-based drugs. Approved immune cell drugs are predominantly CAR-T products for hematological malignancies, originating mainly from the US and China. Approved stem cell drugs are primarily HSC and MSC products, authorized in the US, Japan, South Korea, and China, covering indications such as graft-versus-host disease. Other somatic cell therapies are established for skin repair, bone disorders, and ophthalmology, with South Korea leading in skin-related products.

**Discussion:**

These findings reveal distinct regional strengths and strategic emphases in cell therapy development. The observed patterns are significantly influenced by heterogeneous regulatory frameworks across regions. The global industry is advancing the translation of cell therapy from cutting-edge technology to accessible clinical application through the synergy of scientific innovation and evolving regulatory pathways.

## 1 Introduction

With advancements in biomedical technology, cell therapy has successfully transitioned from theory to clinical practice (Geyer, 2019). Compared to traditional small molecules and biologics, cell therapies offer unique clinical advantages. Living cells can simultaneously respond to systemic and local chemical, physical, and biological cues, readily across biological barriers, and target specific cell types and tissues. They can dynamically react to biological signals and treat diseases by attacking cancer cells, regenerating tissues, and restoring lost biological functions (Jain, 2016).

Based on cell type, cell therapies are categorized into immune cell therapy, stem cell therapy, and other somatic cell therapy. Immune cell therapy exerts therapeutic effects by enhancing immune recognition, strengthening immune attacks, and overcoming immunosuppression. They have achieved significant progress in oncology, infections, and immune disorders (Weber et al., 2020). Stem cells are renewable cells with the potential to differentiate into multiple lineages (Zakrzewski et al., 2019). They can treat diseases by repairing damaged tissues, promoting regeneration, exerting anti-inflammatory and anti-fibrotic effects, and are now widely used in cardiovascular, neurological, immune, and metabolic diseases (Gottipamula et al., 2018; Trounson and DeWitt, 2016). Other somatic cell therapies include islet cell, chondrocyte, and hepatocyte transplantation, functioning through mechanisms like direct replacement of damaged cells, tissue repair and regeneration, and metabolic regulation, playing therapeutic roles in diabetes, liver diseases, orthopedic diseases, etc. (Soria et al., 2001; Forbes et al., 2015; Zellner et al., 2017). Depending on the presence of genetic modification, cell-based medicinal products can be categorized as either *ex vivo* genetically modified or non-genetically modified. This categorization is frequently employed within the national regulatory framework.

While the efficacy of cell therapy is widely recognized in multiple disease areas, and both approved products and clinical applications continues to expand, challenges remain during clinical trials, including imperfect regulatory systems and lack of unified international standards. In this review, we summarize the current status of clinical research in cell therapy, highlight marketed cell therapy products. By analyzing domestic and international regulatory models, we discuss challenges and recommendations in clinical translation, providing a reference for researchers in this field.

## 2 Overview of clinical trial development

**FIGURE 1:**
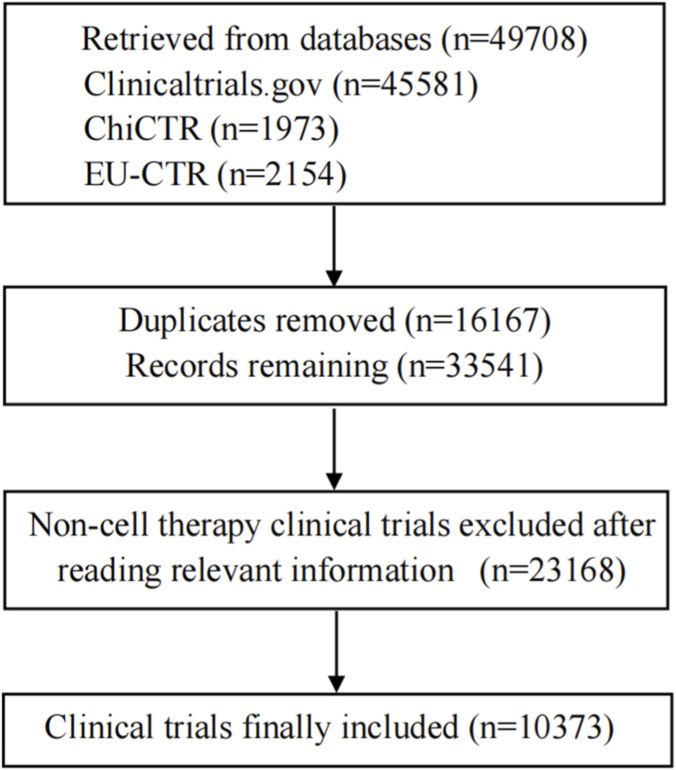
Flowchart of the clinical trial selection process. This diagram outlines the identification and screening steps used to define the final cohort of cell therapy clinical trials analyzed in this study. The initial search across three registries (ClinicalTrials.gov, Chinese Clinical Trial Registry [ChiCTR], and EU Clinical Trials Register [EU-CTR]) yielded a total of 49,708 records. After removing 16,167 duplicate entries, 33,541 unique records remained. These records were then manually screened based on titles, intervention descriptions, and other relevant registry fields. A total of 23,168 records were excluded as they did not pertain to cell therapy interventions (e.g., small molecule, antibody, or non-cellular gene therapies). The final dataset included 10,373 unique cell therapy clinical trials for quantitative and qualitative analysis. The term “reading relevant information” refers to the manual review of registry entries to ascertain whether the primary intervention involved the administration or engineering of living human cells.

As of Octorber 31, 2025, a search of the U.S. ClinicalTrials.gov, the Chinese Clinical Trial Registry (ChiCTR), and the EU Clinical Trials Register (EU-CTR) using the keywords “cell therapy”, “T cell”, “stem cell”, “CAR”, “TCR”, “TIL”, “DC”, and “NK”, returned 32,380 records. After reviewing the trial titles and intervention descriptions, 10,373 were confirmed as cell therapy clinical trials (Figure 1).

**FIGURE 2:**
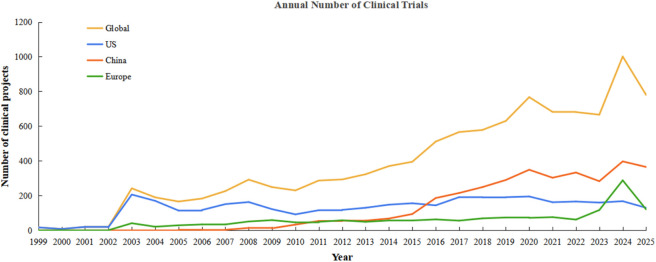
Global clinical trial activity has shown sustained growth from 1999 to 2025, with varying trajectories across major regions. The line chart depicts the annual number of registered clinical trials worldwide and in three key regions: the United States, China, and the European Union (EU), between 1999 and October 2025. The yellow line represents the global total; the blue, red, and green lines represent trials conducted in the United States, China, and the EU, respectively. Data were obtained through a comprehensive search of three major trial registries: ClinicalTrials.gov, the Chinese Clinical Trial Registry (ChiCTR), and the EU Clinical Trials Register (EU-CTR).

Of the 10,373 cell therapy clinical trials, the United States accounted for the largest number (3,563), followed by China in second place (3,365), and Europe collectively had 1,584. According to our dataset, the U.S. was the first to launch a cell therapy clinical trial in 1999 and remained the only country conducting such trials through 2001. China entered this field later, initiating cell therapy trials in 2005, but the number of projects has shown an overall upward trend, surpassing the US in 2016 to hold the world’s highest number of active cell therapy clinical trials (Figure 2).

Among the global 10,373 clinical trials, “Completed” studies had the highest proportion. Both the US and Europe demonstrated completion rates higher than the global average, whereas China’s completion rate was comparatively lower. However, China and Europe had higher proportion of “Ongoing” trials than the US, indicating particularly active clinical research in cell therapy. The proportion of clinical trials that were suspended or terminated was significantly lower in China than in the United States and Europe, a difference that was statistically significant (χ^2^ = 1226.777, P < 0.001). In terms of trial phase, Phase I-II trials predominated globally, reflecting that most trials still remain at early stages. Europe, however, had a significantly greater proportion of Phase III trials compared to China, the US, and the global average (χ^2^ = 1755.168, P < 0.001). Regarding study type, interventional trials accounted for over 70% of studies in every region, with observational studies and expanded access comprising only a minority. The proportion of interventional trials in US substantially exceeded that of China, Europe, and the global average (χ^2^ = 1581.054, P < 0.001). The analysis of study design revealed that single-arm and sequential designs were more common in the US and China than in Europe, whereas Europe led in parallel and factorial designs. This divergence in trial-design focus between Europe, China and the US was also statistically significant (χ^2^ = 719.420, P < 0.001) (Table 1).

**TABLE 1:** Basic characteristics of clinical trials.

Category	Subcategory	Global	%	US	%	China	%	Europe	%	*χ* ^*2*^	*P*
Status	Completed	3547	34.2%	1477	41.5%	982	29.2%	556	35.1%	1226.777	<0.001
	Active, not recruiting/Recruiting	3684	35.5%	1187	33.3%	1310	38.9%	653	41.2%		
	Suspended/Terminated/Withdrawn	1299	12.5%	766	21.5%	127	3.8%	150	9.5%		
	Unknown status/Other	1843	17.8%	133	3.7%	946	28.1%	225	14.2%		
Phase	Early phase I	887	8.6%	61	1.7%	710	21.1%	8	0.5%	1755.168	<0.001
	Phase I	2939	28.3%	1263	35.4%	934	27.8%	267	16.9%		
	Phase I/II	2142	20.6%	590	16.6%	641	19.0%	415	26.2%		
	Phase II	2143	20.7%	1079	30.3%	323	9.6%	435	27.5%		
	Phase II/III	159	1.5%	35	1.0%	31	0.9%	42	2.7%		
	Phase III	435	4.2%	158	4.4%	54	1.6%	166	10.5%		
	Phase IV	110	1.1%	21	0.6%	44	1.3%	24	1.5%		
	Unknown phase	1558	15.0%	356	10.0%	628	18.7%	227	14.3%		
Type	Observational	764	7.4%	125	3.5%	403	12.0%	118	7.4%	1581.054	<0.001
	Interventional	9180	88.5%	3392	95.2%	2934	87.2%	1129	71.3%		
	Expanded access	66	0.6%	46	1.3%	1	0.0%	1	0.1%		
Design	Single arm	368	3.5%	0	0.0%	32	1.0%	336	21.2%	719.420	<0.001
	Parallel assignment	5551	53.5%	1904	53.4%	2107	62.6%	575	36.3%		
	Sequential assignment	2659	25.6%	854	24.0%	783	23.3%	424	26.8%		
	Crossover assignment	682	6.6%	316	8.9%	223	6.6%	52	3.3%		
	Factorial assignment	78	0.8%	32	0.9%	6	0.2%	14	0.9%		
	Unknown design	46	0.4%	10	0.3%	15	0.4%	8	0.5%		

**FIGURE 3:**
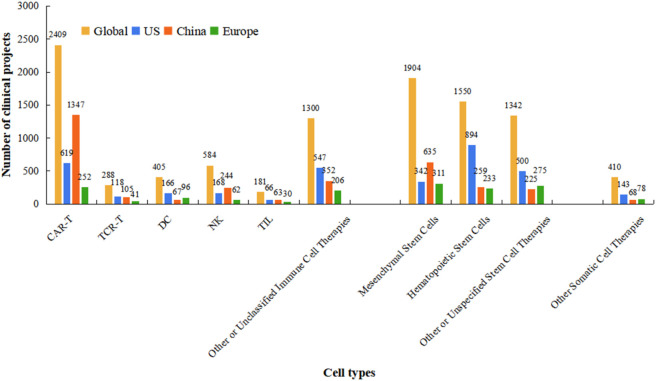
Distribution of clinical trials by cell therapy type across major regions. This grouped bar chart compares the absolute number of registered clinical trials for different categories of cell therapy (e.g., CAR-T, TCR-T, stem cells, etc.) conducted globally and within the United States, China, and the European Union. The data scope and time period (1999 – October 2025) are identical to those in Figure 1. For each cell therapy type on the x-axis, the grouped bars (from left to right within each cluster) represent the trial counts for the Global total, United States, China, and European Union, respectively. The color scheme is consistent with Figure 2: yellow (Global), blue (United States), red (China), and green (European Union). Data were aggregated from the same three registries (ClinicalTrials.gov, ChiCTR, EU-CTR).

**FIGURE 4:**
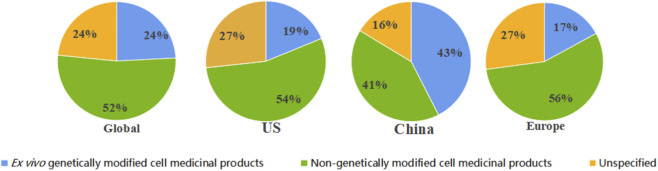
Global and regional profiles of gene modification in cell therapy trials. This panel of four pie charts illustrates the proportion of clinical trials that utilize genetically modified cell products across different regions for the period 1999 to October 2025. Each chart represents a distinct geographic scope: A, Global; B, United States; C, China; D, European Union. Within each pie chart, the segments are categorized based on the gene modification status of the cell therapy: green segments represent non-genetically modified cell therapies; blue segments represent *ex vivo* genetically modified cell therapies; yellow segments represent trials for which this information was unclear or not reported in the registry entry and thus could not be definitively classified. Data were aggregated from the same three registries (ClinicalTrials.gov, ChiCTR, EU-CTR).

When classified by cell therapy type, immune cell therapies accounted for 5,167 trials, and stem cell therapies for 4,796, with similar quantities. CAR-T therapy had the highest number of trials (2,409), accounting for 46.6% of immune cell therapy studies. This was followed by mesenchymal stem cell (MSC) and hematopoietic stem cell (HSC) therapies comprised 1,904 trials (39.7% of stem cell studies) and 1,550 trials (32.3%), respectively. Somatic cell therapy projects were comparatively fewer, with only 410 trials. At national level, research priorities differ. China had more clinical trials in CAR-T, NK cells, and MSCs than the US, with CAR-T sutdies far exceeding those in the US, whereas the US had significantly more HSC trials than China (Figure 3). When categorized by the presence of genetic modification, the number of non-genetically modified cell therapy projects far exceeded that of genetically modified ones in both the United States and Europe. In contrast, no significant difference was observed between the two categories in China (Figure 4).

In terms of indications, cell therapy clinical trials cover a wide range of fields, yet 65% are concentrated in oncology and immune system disorders. Globally, oncology trials accounted for over half (56.1%) of cell therapy trials. Both China and the US had significantly exceed this global average, reaching 66.5% and 61.9% respectively. In contrast, Europe conducts a smaller share of oncology trials but a significantly higher proportion of studies in immune system disorders, circulatory diseases, and musculoskeletal conditions compared with China, the United States, and the global average (Table 2).

**TABLE 2:** Indications for cell therapy clinical trials.

Indication	Global	%	US	%	China	%	Europe	%
Neoplasms	5817	56.1%	2368	66.5%	2084	61.9%	772	48.7%
Immune system diseases	965	9.3%	252	7.1%	291	8.6%	197	12.4%
Circulatory system diseases	482	4.6%	123	3.5%	88	2.6%	125	7.9%
Musculoskeletal system diseases	420	4.0%	93	2.6%	73	2.2%	78	4.9%
Blood or hematopoietic organ diseases	346	3.3%	151	4.2%	111	3.3%	56	3.5%
Respiratory system diseases	328	3.2%	71	2.0%	101	3.0%	48	3.0%
Genitourinary system diseases	348	3.4%	73	2.0%	119	3.5%	59	3.7%
Nervous system diseases	347	3.3%	87	2.4%	111	3.3%	30	1.9%
Digestive system diseases	321	3.1%	68	1.9%	100	3.0%	64	4.0%
Endocrine, nutritional, metabolic diseases	290	2.8%	76	2.1%	78	2.3%	48	3.0%
Infectious diseases	227	2.2%	81	2.3%	75	2.2%	42	2.7%
Skin diseases	168	1.6%	40	1.1%	40	1.2%	26	1.6%
Visual system diseases	143	1.4%	34	1.0%	39	1.2%	20	1.3%
Injury	58	0.6%	15	0.4%	26	0.8%	4	0.3%
Mental, behavioral, neurodevelopmental disorders	31	0.3%	8	0.2%	6	0.2%	3	0.2%
General symptoms and signs	18	0.2%	6	0.2%	3	0.1%	2	0.1%
Ear or mastoid process diseases	5	0.0%	2	0.1%	2	0.1%	0	0.0%
Unspecified	59	0.6%	15	0.4%	18	0.5%	10	0.6%

## 3 Overview of marketed cell therapies and regulations in the United States

There are nearly 30 cell therapy products commercially available in the United States, comprising 19 somatic cell therapies and 9 stem cell therapies. Among these, 15 are autologous and 13 are allogeneic in origin. Furthermore, 12 are genetically modified, while 16 are non-genetically modified (Table 3). In 2010, the FDA approved the first cell therapy product, Sipuleucel-T (brand name Provenge®). Sipuleucel-T, a therapeutic cancer vaccine approved in the U.S. in 2010, demonstrated significant survival benefits in clinical trials. Patients with asymptomatic or minimally symptomatic metastatic castration-resistant prostate cancer treated with Sipuleucel-T showed a 10% higher survival rate at 3 years compared to those receiving placebo (Cheever and Higano, 2011).

**TABLE 3:** Approved cell therapy products in the United States.

Standard name	Brand name	Manufacturer	Approved countries	Approval year	Cell-derived	Cell type	Genetic modification
Sipuleucel-T	Provenge®	Dendreon corporation	United States, EU	2010	Autologous	Somatic cell	No
Azficel-T	LAVIV®	Fibrocell technologies, Inc	United States	2011	Autologous	Somatic cell	No
HPC, cord blood	Hemacord®	New York blood center, Inc	United States	2011	Allogeneic	Stem cells	No
Allogeneic cultured keratinocytes and fibroblasts in bovine collagen	GINTUIT®	Organogenesis incorporated	United States	2012	Allogeneic	Somatic cell	No
HPC, cord blood	Ducord®	Duke university school of medicine	United States	2012	Allogeneic	Stem cells	No
Autologous cultured chondrocytes	MACI®	Vericel Denmark ApS/Vericel corporation	United States	2013	Autologous	Somatic cell	No
HPC, cord blood	ALLOCORD®	SSM cardinal glennon Children’s medical center	United States	2013	Allogeneic	Stem cells	No
HPC, cord blood	Clevecord®	Cleveland Cord blood center	United States	2016	Allogeneic	Stem cells	No
Tisagenlecleucel	Kymriah®	Novartis	United States, EU, Japanetc.	2017	Autologous	Somatic cell	Yes
Axicabtagene ciloleucel	Yescarta®	Kite pharma/Fosun pharma	United States, EU, China, Japanetc.	2017	Autologous	Somatic cell	Yes
Brexucabtagene autoleucel	Tecartus®	Kite pharma	United States, EU	2020	Autologous	Somatic cell	Yes
Lisocabtagene maraleucel	Breyanzi®	Bristol myers squibb	United States, EU	2021	Autologous	Somatic cell	Yes
Idecabtagene vicleucel	Abecma®	Bristol myers squibb	United States, EU	2021	Autologous	Somatic cell	Yes
Allogeneic cultured keratinocytes and dermal fibroblasts in murine collagen (dsat)	Stratagraft®	Stratatech corporation	United States	2021	Allogeneic	Somatic cell	No
Allogeneic processed thymus tissue-agdc	RETHYMIC®	Enzyvant therapeutics GmbH	United States	2021	Allogeneic	Somatic cell	No
Ciltacabtagene autoleucel	Carvykti®	Legend biotech/Johnson and Johnson	United States, EU, Chinaetc.	2022	Autologous	Somatic cell	Yes
Omidubicel-onlv	Omisirge®	Gamida cell	United States	2023	Allogeneic	Stem cells	No
Exagamglogene autotemcel	Casgevy®	Vertex pharmaceuticals incorporated	United States, EU	2023	Autologous	Stem cells	Yes
Lovotibeglogene autotemcel	Lyfgenia®	Bluebird bio	United States	2023	Autologous	Stem cells	Yes
Donislecel	LANTIDRA®	CellTrans Inc	United States	2023	Allogeneic	Somatic cell	No
Obecabtagene autoleucel	Aucatzyl®	Autolus therapeutics	United States	2024	Autologous	Somatic cell	Yes
Lifileucel	Amtagvi®	Iovance biotherapeutics	United States	2024	Autologous	Somatic cell	No
Afamitresgene autoleucel	Tecelra®	Adaptimmune LLC	United States	2024	Autologous	Somatic cell	Yes
Remestemcel-L-rknd	Ryoncil®	Mesoblast	United States	2024	Allogeneic	Stem cells	No
Acellular tissue engineered vessel-tyod	SYMVESS®	Humacyte global, Inc	United States	2024	Allogeneic	Somatic cell	No
HPC, cord blood	REGENECYTE®	StemCyte, Inc	United States	2024	Allogeneic	Stem cells	No
Revakinagene taroretcel	ENCELTO®	Neurotech Pharmaceuticals,Inc	United States	2025	Allogeneic	Somatic cell	Yes
Prademagene zamikeracel	ZEVASKYN®	Abeona therapeutics, Inc	United States	2025	Autologous	Somatic cell	Yes

Hemacord® was the first hematopoietic stem cell therapy product approved by the U.S. FDA. Subsequently, several other hematopoietic stem cell products such as Ducord®, ALLOCORD®, and Clevecord® have been approved, establishing global leadership in this field (Voelker, 2011). Among genetically modified hematopoietic stem cell products, Exagamglogene autotemcel (brand name Casgevy®) has shown remarkable efficacy in treating recurrent vaso-occlusive crises (VOC) in patients aged 12 years and older with sickle cell disease. Results from a Phase III clinical trial involving 44 patients treated with Exagamglogene autotemcel, followed for 19.3 months, indicated that 29 out of 30 adequately followed patients remained free of VOC for at least 12 consecutive months, and all 30 patients avoided VOC-related hospitalizations for the same period. The treatment eliminated VOC in 97% of sickle cell disease patients for a duration of at least 1 year (Frangoul et al., 2024). This drug is also under clinical investigation for thalassemia, showing promising results with 91% of transfusion-dependent β-thalassemia patients achieving transfusion independence, although this indication has not yet been approved (Locatelli et al., 2024).

Lovotibeglogene autotemcel (brand name Lyfgenia®) was approved in 2023 for the treatment of sickle cell disease (SCD) patients aged ≥12 years with a history of recurrent vaso-occlusive events (VOE). Clinical trials demonstrated its favorable efficacy and safety profile. Among 47 treated patients, 33 were evaluable. Of these, 30 experienced complete resolution of VOE and severe VOE within 6–18 months post-infusion. Hemolytic markers in all 33 patients approached normal levels, and patients reported sustained improvements in symptoms such as pain intensity and fatigue (Kanter et al., 2023). Additionally, Omidubicel-onlv (brand name Omisirge®), a non-genetically modified allogeneic hematopoietic stem cell therapy product, was approved in the U.S. in 2023. Clinical trial results demonstrated its efficacy in treating hematologic malignancies. In a study conducted in the U.S. from 2020 to 2023 involving 36 enrolled patients, 29 received Omidubicel-onlv transplants. Two patients experienced graft failure. The disease-free survival (DFS) rates at 100 days and 365 days post-transplant were 95.2% and 82.5%, respectively, while the overall survival (OS) rates were 96.3% and 84.2% (Horwitz et al., 2023).

While the United States leads globally in the hematopoietic stem cell domain, only one mesenchymal stem cell product, Remestemcel-L-rknd (brand name Ryoncil®), has been approved, with its market entry in 2024 (Kurtzberg et al., 2020).

Immune cell therapy is a major global research focus. The United States has approved seven CAR-T products, leading in quantity worldwide. In 2012, Novartis’s Tisagenlecleucel (brand name Kymriah®) achieved remarkable results in treating a 6-year-old child with acute lymphoblastic leukemia, marking the first widely reported case of a patient cured by CAR-T therapy and remaining relapse-free over a 10-year follow-up (Awasthi et al., 2023). A clinical trial involving 59 patients with relapsed/refractory lymphoblastic leukemia demonstrated an overall response rate (ORR) of 93% 1 month after Tisagenlecleucel infusion, highlighting its significant efficacy (Maude et al., 2016). A 3-year follow-up of patients treated with Tisagenlecleucel for relapsed/refractory lymphoblastic leukemia showed progression-free survival (PFS) and overall survival (OS) rates of 44% and 63%, respectively, indicating favorable long-term safety (Laetsch et al., 2023). Based on its outstanding performance, Kymriah® became the first CAR-T therapy approved globally, receiving U.S. approval in 2017.

Kite Pharma introduced two CAR-T therapy products in October 2017 and July 2020, respectively. The 2017 product, Yescarta®, demonstrated an ORR of 82% and a complete response (CR) rate of 54% in 101 patients with refractory large B-cell lymphoma, with an 18-month OS rate of 52% (Neelapu et al., 2017; Locke et al., 2019). In 2020, another CAR-T therapy from Kite Pharma, Brexucabtagene autoleucel (brand name Tecartus), was approved. Clinical trials for mantle cell lymphoma showed a high CR of 67% among 76 patients, with 12-month PFS and OS rates of 61% and 83%, respectively (Wang et al., 2020).

In 2021, Bristol Myers Squibb launched two CAR-T therapies: Lisocabtagene maraleucel (brand name Breyanzi®) and Idecabtagene vicleucel (brand name Abecma®). Lisocabtagene maraleucel demonstrated favorable efficacy in treating relapsed/refractory large B-cell lymphoma and relapsed/refractory follicular lymphoma (Abramson et al., 2020; Morschhauser et al., 2024). Compared to standard therapy, it increased ORR by 31% and reduced adverse event (AE) incidence by 3% in relapsed/refractory large B-cell lymphoma patients, showing superior efficacy and safety (Abramson et al., 2023). Currently, the efficacy and safety of Lisocabtagene maraleucel for relapsed/refractory mantle cell lymphoma and relapsed/refractory chronic lymphocytic leukemia/small lymphocytic lymphoma are under investigation. Idecabtagene vicleucel was approved in 2021 for relapsed or refractory multiple myeloma after ≥4 prior lines of therapy. Due to its strong performance in Phase III trials, it received approval for second-line therapy in 2024 (Sharma et al., 2025).

Ciltacabtagene Autoleucel (brand name Carvykti®) is a CAR-T cell therapy co-developed by the Chinese innovative pharmaceutical company Legend Biotech and Janssen, part of Johnson and Johnson. This drug demonstrated remarkable efficacy in treating relapsed/refractory multiple myeloma, with an ORR of 97.9%, a CR of 82.5%, and 27-month PFS and OS rates of 54.9% and 70.4%, respectively (Berdeja et al., 2021; Martin et al., 2023). Carvykti® received approvals from the U.S. FDA, the European EMA, and the Japanese PMDA in 2022.

Obecabtagene autoleucel (brand name Aucatzyl®) was approved by the FDA in 2024. It is notable for its favorable safety profile, with Grade 3 or higher cytokine release syndrome occurring in 2.4% of patients and Grade 3 or higher immune effector cell-associated neurotoxicity syndrome in 7.1%. Given its safety, it is suitable for outpatient use (Roddie et al., 2024).

In TIL therapy, the innovative drug Lifileucel (brand name Amtagvi®), developed by Iovance Biotherapeutics, was approved in the U.S. in 2024. It is currently the only commercially available TIL therapy globally, indicated for the treatment of adults with unresectable or metastatic melanoma. Clinical trial results demonstrated breakthrough efficacy in melanoma treatment. Among 153 patients treated, the ORR was 31.4%, with 79.3% showing reduced tumor burden. Four patients achieved a complete response (CR) 1 year post-treatment. The 5-year OS rate was 19.7%, and 31.3% of responding patients maintained a CR or partial response (PR) status after 5 years (Medina et al., 2025).

Afamitresgene autoleucel (brand name Tecelra®) is the first approved TCR-T therapy in US. Results from its Phase II clinical trial involving 52 patients with synovial sarcoma and myxoid/round cell liposarcoma showed an ORR of 43%, with a median duration of response of 6 months. Among responders, 39% maintained their response for 12 months or longer. The most common adverse event was cytokine release syndrome, with only one case reaching Grade 3 (D'Angelo et al., 2024).

Beyond immune cell therapies, the United States has approved numerous products in other somatic cell therapy domains. In dermatology, Azficel-T (brand name LAVIV®) was approved in 2011 for subcutaneous tissue repair and augmentation. A large randomized double-blind clinical trial involving 372 patients demonstrated its significant efficacy, and subsequent studies have shown its benefit in treating vocal cord scars (Smith et al., 2012; Ma et al., 2020). Stratagraft® was approved in 2021. Clinical trial results in burn patients indicated that no significant differences in observer total and overall opinion POSAS scores were observed between StrataGraft tissue and autograft treatment sites at any timepoint. StrataGraft tissue treatment of deep partial-thickness thermal burns reduced the need for autograft, resulted in wound closure and treatment-site cosmesis comparable to that of autograft, and was well tolerated (Holmes et al., 2019).

Autologous cultured chondrocytes (brand name MACI®) were approved in 2013 for repairing symptomatic cartilage defects in the adult knee. A study of 65 patients treated with MACI® showed the median Tegner score improved from II to IV at 12 months, an improvement maintained to 60 months. The mean Lysholm score improved from 28.5 to 76.6 points (±19.8) at 24 months.

Allogeneic processed thymus tissue-agdc (brand name GINTUIT®) is a therapy for congenital athymia (CA), an immune deficiency disorder that increases patient mortality risk from infections, autoimmune diseases, or cancer. This drug is an effective treatment for CA. Clinical trial results involving 34 patients showed no surgery-related adverse events post-transplant. Evidence of immune reconstitution was present in 45% and 91% of patients at 6 and 12 months, respectively. The 1-year OS rate was 94%. Although 50% of patients still experienced developmental delays, allogeneic processed thymus tissue-agdc provides a new treatment option for CA patients (Alqahtani et al., 2025).

Donislecel (brand name LANTIDRA®) is a therapy designed to help patients with type I diabetes restore glycemic control, eliminate hypoglycemic episodes, and reduce or eliminate exogenous insulin injections. In a study of 30 patients treated with donislecel, 19 achieved HbA1c ≤ 6.5% with no severe hypoglycemic events in the year following their last transplant. Twenty patients were insulin-independent 1 year after their last transplant, with some maintaining good glycemic control for up to 6 years, significantly improving quality of life (RIOS et al., 2024).

Acellular tissue engineered vessel-tyod (brand name SYMVESS®) is indicated for urgent vascular reconstruction in adult limb arterial injuries, serving as a vascular conduit to reduce amputation rates when limb salvage is at risk and autologous vein graft is not feasible. A clinical trial across 24 medical sites in the U.S., Israel, and Ukraine involving 67 patients showed a secondary patency rate of 91.5%, an amputation rate of 4.5%, and a mortality rate of 3.5% at 30 days post-treatment (Moore et al., 2025). In 2025, Fulton F. Velez et al. developed a model incorporating SYMVESS®, estimating that its use could reduce amputations by 29.8%, graft infections by 29.5%, and treatment costs by 16.7% over 3 years, demonstrating significant patient benefit (Velez et al., 2025).

Revakinagene taroretcel (brand name ENCELTO®) is used to treat chronic retinal diseases. Clinical studies indicate its efficacy in treating conditions such as macular telangiectasia, glaucoma, and retinitis pigmentosa (Hoy, 2025).

Prademagene zamikeracel (brand name ZEVASKYN®) is a new drug approved in 2025 for treating recessive dystrophic epidermolysis bullosa. In a clinical trial involving 86 wounds in patients with this condition, prademagene zamikeracel increased the wound healing rate by 67% compared to the control group at 24 weeks, with no serious adverse events observed (Tang et al., 2025).

The United States established its regulatory framework for cell therapies early, with the FDA releasing the “Guidance on Human Cells and Tissues” in 1993. Currently, the FDA oversees cell therapy products primarily under the Federal Food, Drug, and Cosmetic Act and the Public Health Service Act, regulating them as biological products. This is managed by the FDA’s Center for Biologics Evaluation and Research and the Office of Cellular Therapy and Gene Therapy.

Cell therapy products in the U.S. are categorized as either 351 products or 361 products. Products involving “more than minimal manipulation” (e.g., changes in homologous use, *in vitro* cultivation and expansion, genetic modification) or intended for “non-homologous use” are defined as “351 products” (biological products/drugs) and are subject to the strictest regulations, requiring Investigational New Drug (IND) and Biologics License Application (BLA) submissions. Products undergoing only “minimal manipulation” (e.g., separation, washing, freezing) and intended for “homologous use” are regulated as “361 products” (Human Cells, Tissues, and Cellular and Tissue-Based Products) under lighter oversight, with certain exemptions during approval. Genetically modified products are regulated under the highest standards regardless of origin.

Regarding approval mechanisms, the U.S. has established the Regenerative Medicine Advanced Therapy (RMAT) pathway, allowing conditional approval based on Phase II clinical trial data. For example, Ryoncil® was first submitted for approval in 2019, rejected in 2020, and rejected again in 2023. It was ultimately approved in 2024 after supplementary data were provided following an agreement reached at the end of 2023. While maintaining rigorous evidence requirements, the FDA demonstrates scientific flexibility through close communication with companies to develop feasible solutions (Franco et al., 2023).

## 4 Overview of marketed cell therapies and regulations in the China

There are 8 cell therapy products approved for marketing in China (Table 4). Among them, two were co-developed by domestic and international companies: one is the localized version of Yescarta®, introduced in China through a collaboration between Kite Pharma and Fosun Pharmaceutical, and the other is Ciltacabtagene Autoleucel (brand name Carvykti®), jointly developed by Legend Biotech and Janssen, part of Johnson and Johnson. Additionally, there are 6 cell therapy products independently developed in China, five of which are CAR-T therapies, and one is a stem cell therapy. Due to China’s relatively late start in cell therapy research, all these products were approved in recent years.

**TABLE 4:** *A*pproved cell therapy products in the China.

Standard name	Brand name	Manufacturer	Approved countries	Approval year	Cell-derived	Cell type	Genetic modification
Relmacabtagene autoleucel	Carteyva®	JW therapeutics	China	2021	Autologous	Somatic cell	Yes
Equecabtagene autoleucel	Fucaso®	IASO biotherapeutics/Innovent biologics	China, South Korea	2023	Autologous	Somatic cell	Yes
Inaticabtagene autoleucel	Yuanruida®	Heyuan biotechnology	China	2023	Autologous	Somatic cell	Yes
Zevorcabtagene autoleucel	Saikaize®	CARsgen therapeutics/Huadong medicine	China	2024	Autologous	Somatic cell	Yes
Renikieleucel injection	Hengkailai®	Hengrun dasheng	China	2025	Autologous	Somatic cell	Yes
Amimestrocel injection	Ruibosheng®	Biosun excellence biotech	China	2025	Allogeneic	Stem cells	No

In 2021, Relmacabtagene autoleucel (brand name Carteyva®), developed by the domestic company JW Therapeutics, demonstrated significant efficacy in clinical trials for the treatment of certain types of non-Hodgkin’s lymphoma and was approved in China as a Class 1 biological product. It is the first CAR-T therapy independently developed and approved by a Chinese company (Song et al., 2021; Ying et al., 2022; Song et al., 2022).

In 2023, Equecabtagene Autoleucel, co-developed by IASO Bio and Innovent Biologics, was successfully approved in China. Clinical trial results showed that among 103 subjects with relapsed/refractory multiple myeloma, only one experienced a Grade 3 adverse reaction, manifested as cytokine release syndrome, which resolved after treatment (Li et al., 2024).

In 2023, Inaticabtagene Autoleucel (brand name Yuanruida®), developed by the Chinese company Hebei Senlang Biotechnology Inc. (HeCel), was approved in China. This drug is the first CAR-T cell therapy product approved for leukemia treatment in China and has shown promising results in clinical trials for pediatric leukemia (Zheng et al., 2024). A clinical trial involving Chinese patients aged 3–18 with relapsed/refractory B-cell acute lymphoblastic leukemia showed that among 12 patients receiving Inaticabtagene Autoleucel infusion, all achieved an overall response rate (ORR), with 5 achieving complete response (CR). Among Grade ≥3 adverse reactions, one case was cytokine release syndrome and two were immune effector cell-associated neurotoxicity syndrome; all adverse reactions resolved after management without sequelae (Wang et al., 2024). Inaticabtagene Autoleucel is currently the lowest-priced marketed CAR-T therapy. Its inclusion in China’s national medical insurance scheme, at a cost of $140,035, significantly alleviates the financial burden on patients (Zheng and Zhang, 2024).

Zevorcabtagene Autoleucel (brand name Saikaize®), co-developed by CARsgen Therapeutics and Huadong Medicine, was approved in China in 2024. This drug has shown favorable survival benefits. A follow-up study of 14 patients with relapsed/refractory multiple myeloma treated with Zevorcabtagene Autoleucel revealed a median progression-free survival (PFS) of 25 months and a median duration of response of 24.1 months, demonstrating deep and durable efficacy over approximately 3 years of follow-up (Fu et al., 2023).

Renikieleucel was evaluated in a Phase II clinical trial involving 81 patients with non-Hodgkin’s lymphoma (NHL). Results showed ORRs of 53.1%, 45.7%, and 74.1% at 3 months, 6 months, and best response, respectively. Complete response rates (CRR) were 32.1%, 29.6%, and 49.4% at the same time points. The median duration of response (DOR) was 339 days, and median PFS was 176 days. Renikieleucel demonstrated excellent efficacy and manageable safety in clinical trials. The approval of this product provides a new treatment option for patients with relapsed/refractory large B-cell lymphoma (Zhang et al., 2024).

Amimestrocel Injection (brand name Ruibosheng®) is China’s first mesenchymal stem cell therapy. It has shown significant efficacy in clinical trials for treating acute graft-versus-host disease (aGVHD) in patients aged 14 and older, primarily with gastrointestinal involvement, who have failed corticosteroid therapy. Results from a multicenter, randomized, double-blind, placebo-controlled clinical trial involving 78 patients (40 in the treatment group, 38 in the control group) showed that the ORR on day 28 was significantly higher in the treatment group compared to the control group (71.9% vs. 46.7%, P = 0.043). The 2-year cumulative incidence of non-response was slightly lower in the treatment group (16.5% vs. 46.7%, P = 0.056), with no significant difference in adverse reaction rates between the two groups (Jiang et al., 2024). Given China’s later start in cell therapy research, all domestically developed cell therapy products have been approved in recent years.

The National Medical Products Administration (NMPA) of China has gradually established a regulatory framework for cell therapy products that aligns with international standards while incorporating Chinese characteristics. China’s management of cell therapies involves separate oversight by drug regulatory authorities and health administrative departments, implementing a dual-track system based on classification as either a new drug or a medical technology. As drugs, they must comply with the Drug Registration Management Measures and be registered as biological products. Genetically modified cell products must also meet requirements outlined in guidelines such as the Technical Guiding Principles for Non-clinical Research of Genetically Modified Cell Therapy Products. The medical technology pathway generally applies to exploratory, non-commercial use of unapproved products within medical institutions, which must undergo strict academic and ethical reviews. This primarily applies to stem cell clinical research and must adhere to regulations such as the Stem Cell Clinical Research Management Measures. Stem cell projects that demonstrate preliminary safety and efficacy within the medical technology pathway are encouraged to transition to the drug development pathway for further development and eventual market application.

Regarding approval mechanisms, the NMPA has joined the International Council for Harmonisation (ICH), aligning its drug review standards with international norms. It has also established procedures such as the “Breakthrough Therapy Designation,” “Conditional Approval,” and “Priority Review” to accelerate the marketing process for cell therapy products intended for serious diseases with urgent clinical needs. China’s national medical insurance negotiation policy has effectively reduced the prices of cell therapies. This strategy enhances patient access but also compresses profit margins for companies, incentivizing them to reduce costs and improve efficiency through technological innovation.

## 5 Overview of marketed cell therapies and regulations in the EU

Over 10 cell therapy products have been approved for marketing in the European Union (Table 5). Among these, products such as Kymriah®, Yescarta®, and Tecartus® were first granted priority approval by the U.S. FDA. Other products like Alofisel® and ChondroCelect® have been withdrawn from the market for various reasons after their initial approval. Among the currently marketed cell therapy products, seven were first approved in the EU. Of these, three products approved in the EU subsequently received FDA approval for marketing in the United States.

**TABLE 5:** *Approved Cell Therapy Products in the EU*.

Standard name	Brand name	Manufacturer	Approved countries	Approval year	Cell-derived	Cell type	Genetic modification
*ex vivo* expanded autologous human corneal epithelial cells containing stem cells	Holoclar®	Holostem s.r.l	EU	2015	Autologous	Somatic cell	No
Spheroids of human autologous matrix-associated chondrocytes	Spherox®	CO.DON GmbH	EU	2017	Autologous	Somatic cell	No
Betibeglogene autotemcel	Zynteglo®	Bluebird bio	EU, United States	2019	Autologous	Stem cells	Yes
Atidarsagene autotemcel	Lenmeldy®	Orchard therapeutics	EU, United States	2020	Autologous	Stem cells	Yes
Elivaldogene autotemcel	Skysona®	Bluebird bio	EU, United States	2021	Autologous	Stem cells	Yes
Tabelecleucel	Eevallo®	Pierre fabre medicament	EU	2022	Allogeneic	Somatic cell	No
UM171 cell therapy	Zemcelpro®	Cordex biologics	EU	2025	Allogeneic	Stem cells	No

All three of these therapies are genetically modified autologous stem cell treatments. Betibeglogene autotemcel (brand name Zynteglo®) is a one-time therapy for patients with transfusion-dependent β-thalassemia (TDT), designed to eliminate transfusion dependence. Clinical trials have confirmed its favorable efficacy. In a study of 23 patients treated with Betibeglogene autotemcel and followed for 29.5 months, 20 achieved transfusion independence. The mean hemoglobin level was 11.7 g/dL during treatment and 8.7 g/dL at 12 months. When follow-up for these 20 patients was extended to 47.9 months, excluding one ineligible patient and one who withdrew consent, all remaining 18 patients maintained transfusion independence, with no reported deaths (Locatelli et al., 2022; Kwiatkowski et al., 2024).

Atidarsagene autotemcel (brand name Lenmeldy®) is a cell therapy for the treatment of metachromatic leukodystrophy (MLD) in children. Compared to untreated patients, those receiving Atidarsagene autotemcel showed a significant reduction in the risk of severe motor impairment or death. In patients with late infantile MLD, the proportion surviving free of severe motor impairment at age 6 was 0% in the untreated group versus 100% in the treated group. At age 10, the corresponding proportions were 11.2% in the untreated group and 87.5% in the treated group (Fumagalli et al., 2025).

Elivaldogene autotemcel (brand name Skysona®) is indicated for the treatment of early active cerebral adrenoleukodystrophy (CALD) in adolescent boys. Clinical trial results showed that after a median follow-up of 29 months in 17 patients treated with Elivaldogene autotemcel, 15 patients survived without major functional disabilities and with minimal clinical symptoms. One patient died due to disease progression, and another, who withdrew from the study and received an allogeneic stem cell transplant, died from transplant-related complications. The efficacy of this drug is internationally recognized, and it is now marketed in both the United States and the European Union (Eichler et al., 2017).

Zemcelpro® represents a significant technological advancement in the field of umbilical cord blood transplantation by amplifying the therapeutic potential of cord blood through an innovative chemical expansion method. Its clinical trial results have also demonstrated efficacy and safety (Cohen et al., 2020).

In the category of non-genetically modified somatic cell therapy products, Tabelecleucel (brand name Ebvallo®) is the world’s first virus-specific T-cell (VST) therapy. Approved in the EU in 2022, it provides a treatment option for patients with relapsed or refractory EBV-positive post-transplant lymphoproliferative disorder. Clinical trial results showed an objective response rate of 51.2% and a significantly prolonged overall survival compared to other therapies (Mahadeo et al., 2024; Barlev et al., 2024).

Beyond immune cell therapies, the EU has approved two additional somatic cell therapy products. In 2015, *ex vivo* expanded autologous human corneal epithelial cells containing stem cells (brand name Holoclar®) was approved. In a study of 16 subjects with severe unilateral limbal stem cell deficiency caused by chemical burns, 10 patients achieved complete recovery at 12 months post-operation, 3 experienced corneal surface instability or partial conjunctival re-growth, and 3 treatments failed. This therapy can help restore vision in patients with corneal damage (Marchini et al., 2012).

Spheroids of human autologous matrix-associated chondrocytes (brand name Spherox®), approved in 2017, has been proven to yield higher Knee Injury and Osteoarthritis Outcome Scores and lower pain indices with statistical significance compared to conventional materials in the treatment of knee cartilage defects (Bumberger et al., 2024).

The European Medicines Agency (EMA) is primarily responsible for the scientific evaluation, supervision, and coordination of marketing authorizations in the EU. Its Committee for Advanced Therapies (CAT), composed of experts in gene therapy, cell therapy, tissue engineering, and medical ethics, provides core scientific opinions on development, classification, evaluation, and monitoring.

The regulatory framework for cell therapy products in the EU primarily follows the Regulation on Advanced Therapy Medicinal Products (ATMP Regulation). This regulation classifies cell therapy products, along with gene therapy and tissue engineering products, as Advanced Therapy Medicinal Products (ATMPs) and establishes unified regulatory rules. A centralized regulatory approach is implemented across EU member states. For a cell therapy product to be marketed and commercially distributed within the EU, it must undergo the centralized procedure by submitting a marketing authorization application to the EMA. Upon approval, the European Commission grants a marketing authorization valid in all EU member states.

Regarding approval mechanisms, support measures such as the Priority Medicines (PRIME) scheme and certification procedures are available to accelerate medicine assessment and reduce the development burden. Furthermore, a unique flexible pathway within the EU allows member states, under specific conditions (e.g., customized production, not manufactured on an industrial scale, hospital-based use), to utilize ATMPs within their national healthcare institutions without obtaining a centralized marketing authorization.

## 6 Overview of marketed cell therapies and regulations in the other country

Apart from the United States, China, and the European Union, Japan and South Korea have also approved a notable number of cell therapy products for market. However, neither country has granted formal market approval to any fully domestically developed genetically modified cell therapy product (Table 6).

**TABLE 6:** Approved cell therapy products in the other country.

Standard name	Brand name	Manufacturer	Approved countries	Approval year	Cell-derived	Cell type	Genetic modification
Human autologous epidermal cell sheet	JACE®	Japan tissue engineering co	Japan	2007	Autologous	Somatic cell	No
Human autologous cartilage cell sheet	JACC®	Japan tissue engineering co	Japan	2012	Autologous	Somatic cell	No
Human allogeneic bone marrow–derived MSC	TEMCELL®	JCR pharmaceuticals	Japan	2015	Allogeneic	Stem cells	No
Autologous bone marrow MSC	Stemirac®	Mesoblast	Japan	2018	Autologous	Stem cells	No
Autologous corneal epithelial cell sheet	Nepic®	Kyoto prefectural univ. Med	Japan	2020	Autologous	Somatic cell	No
Autologous oral mucosal epithelial cell sheet	Ocural®	Kyoto prefectural univ. Med	Japan	2021	Autologous	Somatic cell	No
Melanocyte‐containing epidermal cell sheet	JACEMIN®	Japan tissue engineering co	Japan	2023	Autologous	Somatic cell	No
Allogeneic corneal endothelial cell injection	Vyznova®	Aurion biotech Japan, LLC	Japan	2023	Allogeneic	Somatic cell	No
Autologous chondrocyte spheroids	Chondron®	Sewon cellontech co., Ltd.	South Korea	2001	Autologous	Somatic cell	No
Autologous keratinocyte sheet	Holoderm®	Tego science, Inc.	South Korea	2002	Autologous	Somatic cell	No
Allogeneic keratinocyte sheet	Kaloderm®	Tego science, Inc.	South Korea	2005	Allogeneic	Somatic cell	No
Autologous keratinocyte spray	KeraHeal®	Biosolution co., Ltd.	South Korea	2006	Autologous	Somatic cell	No
Autologous dendritic cell vaccine	CreaVax-RCC Inj	JW CreaGene corp	South Korea	2007	Autologous	Somatic cell	No
Autologous activated lymphocytes	Immuncell-LC®	Innocell co., Ltd.	South Korea	2007	Autologous	Somatic cell	No
Autologous osteocyte implantation	RMS Ossron®	Ossron co., Ltd.	South Korea	2009	Autologous	Somatic cell	No
Autologous adipose stromal vascular fraction (SVF)	QueenCell®	Anterogen co., Ltd.	South Korea	2010	Autologous	Somatic cell	No
Autologous fibroblast implantation	CureSkin®	S.Biomedics co., Ltd.	South Korea	2010	Autologous	Somatic cell	No
Autologous bone marrow MSCs	Cellgram-AMI®	Pharmicell co., Ltd.	South Korea	2011	Autologous	Stem cells	No
Autologous adipose MSCs	Cupistem®	Anterogen co., Ltd.	South Korea	2012	Autologous	Stem cells	No
Allogeneic umbilical MSCs	Cartistem®	Medipost co., Ltd.	South Korea	2012	Allogeneic	Stem cells	No
Autologous bone marrow MSCs	Neuronata-R®	PharmaCell co., Ltd.	South Korea	2014	Autologous	Stem cells	No
Autologous cultured fibroblasts	ROSMIR®	Tego science, Inc.	South Korea	2017	Autologous	Somatic cell	No
Autologous costal chondrocyte spheroids	CartiLife®	Biosolution co., Ltd.	South Korea	2019	Autologous	Somatic cell	No

In the field of mesenchymal stem cell (MSC) therapy, both countries have approved several products. South Korea, with four products approved domestically, holds a leading global position in this area. Research in South Korea began relatively early, with the first MSC product approved as early as 2011. Autologous bone marrow MSCs (brand name Cellgram-AMI®), developed in South Korea for improving cardiac function after acute myocardial infarction, was the world’s first stem cell drug (Fernández-Garza et al., 2023). Results from a randomized controlled clinical trial showed that patients treated with Autologous bone marrow MSCs experienced an increase in left ventricular ejection fraction (LVEF) of 8.8% ± 2.9% after 4 months, compared to an increase of 4.8% ± 1.9% in patients receiving conventional treatment, with significant improvement sustained at the 12-month follow-up (Kim et al., 2018).

Another autologous MSC therapy developed in South Korea, Autologous adipose MSCs (brand name Cupistem®), was approved in 2012 for the treatment of complex anal fistulas in Crohn’s disease. Follow-up of a clinical trial involving 65 patients showed a cumulative fistula closure rate of 66.2% after 1 year, 73.8% after 2 years, and 75.4% after 3 years (Park et al., 2024). In the same year, another stem cell therapy, Allogeneic umbilical MSCs (brand name Cartistem®), was approved in South Korea. This product demonstrated significant efficacy in treating knee cartilage defects in patients with osteoarthritis (ICRS grade IV) caused by degenerative diseases or repetitive trauma. In a Phase III clinical trial involving 114 subjects, 89 completed the treatment regimen, and 73 participated in a 5-year follow-up study. Results indicated a 26% higher improvement rate in patients receiving Allogeneic umbilical MSCs compared to other treatments, with significant pain reduction observed during the 3- to 5-year follow-up period (Lim et al., 2021).

In 2014, Autologous bone marrow MSCs (brand name Neuronata-R®) was approved in South Korea, marking the world’s first stem cell-based therapy for amyotrophic lateral sclerosis (ALS). A clinical trial involving 157 subjects showed that patients treated with Autologous bone marrow MSCs had a higher probability of survival compared to the control group. No serious adverse drug reactions were reported during the 1-year safety assessment period following the first administration (Nam et al., 2023).

Japan has approved two mesenchymal stem cell products. In 2015, Human allogeneic bone marrow–derived MSC (brand name TEMCELL®), developed by JCR Pharmaceuticals, was approved for the treatment of steroid-refractory acute graft-versus-host disease (aGvHD). Results from a multicenter Phase II clinical trial showed that 94% of 32 patients responded to treatment, with 77% achieving complete response (CR) and 16% achieving partial response (PR). No infusion toxicities or ectopic tissue formation were reported (Kebriaei et al., 2009). Human allogeneic bone marrow–derived MSC also showed good efficacy in pediatric aGvHD. A clinical trial involving pediatric patients aged 5–17 showed that among 40 patients who received more than 8 infusions, 57.5% exhibited disease improvement, with 16 patients achieving complete resolution. Among those who achieved an objective response by day 28, 76.1% survived for at least 100 days after the first infusion, demonstrating favorable survival benefit (Kurtzberg et al., 2014).

Mesoblast’s Autologous bone marrow MSC product, Stemirac®, approved in 2018, has shown promising efficacy in treating neurological dysfunction related to spinal cord injury. Clinical trial results indicated that 12 out of 13 patients showed neurological improvement based on the American Spinal Injury Association (ASIA) Impairment Scale at 6 months post-treatment. Among them, 6 ASIA A patients improved to grade B or C, 2 ASIA B patients improved to grade C or D, and 5 ASIA C patients improved to grade D. Notably, these 5 patients showed improvement from grade C to D as early as 1 day after infusion (Honmou et al., 2021).

In the field of somatic cell therapy, approved products are primarily concentrated in dermatology, orthopedics, and ophthalmology. In dermatology, South Korea has developed six products—Holoderm®, Kaloderm®, KeraHeal®, QueenCell®, CureSkin®, and ROSMIR®—mainly for treating skin burns and subcutaneous tissue defects. Japan has two products: JACE® for burns and JACEMIN® for vitiligo, both with clinically proven efficacy. Notably, Japan’s Human autologous epidermal cell sheet (brand name JACE®), supported by substantial data demonstrating efficacy in burn treatment, is expanding its therapeutic applications and is currently undergoing research for mole removal (Morimoto et al., 2018).

In orthopedics, South Korea has three marketed products: Chondron®, RMS Ossron®, and CartiLife®, all of which demonstrated positive results in clinical studies (Choi et al., 2010; Lee, 2018). Among them, Autologous osteocyte implantation (brand name RMS Ossron®), used for local bone formation, is being investigated for conditions such as femoral head necrosis and spinal deformities (Yoon et al., 2024; Kim et al., 2016). Japan has one product in this category, JACC®, which has shown excellent safety in clinical trials (Kato et al., 2024).

In ophthalmology, Japan has launched three products. Two cell therapy drugs for limbal stem cell deficiency were successively approved in 2020 and 2021: Autologous corneal epithelial cell sheet (brand name Nepic®), with an epithelial reconstruction rate of approximately 60%, and Autologous oral mucosal epithelial cell sheet (brand name Ocural®), achieving a rate of 70% (Oie et al., 2023; Cabral et al., 2020). In 2023, Allogeneic corneal endothelial cell injection (brand name Vyznova®) was approved for treating bullous keratopathy, demonstrating a disease improvement rate as high as 88.9% in clinical trials with a favorable safety profile (Holland et al., 2022).

In the field of immune cell therapy, South Korea approved one dendritic cell (DC) vaccine in 2007: Autologous dendritic cell vaccine (brand name CreaVax-RCC Inj.®) for metastatic renal cell carcinoma. Among 9 patients receiving two treatment cycles, 1 achieved partial response (PR), 5 had stable disease (SD), and 3 experienced progressive disease (PD). The median follow-up was 17.5 months, with a median overall survival (OS) of 29 months. The vaccine was well-tolerated with no serious adverse reactions (Kim et al., 2007).

Regarding regulatory policies, Japan was one of the first countries globally to establish a dedicated legal framework for regenerative medicine products, pioneering the world’s first “dual-track” fast-approval pathway specifically for such products. Its regulatory system is recognized for its innovation and foresight. Japan classifies and manages cell therapies based on risk. Products derived from allogeneic sources, genetically modified sources, or iPSCs/ESCs are categorized as high-risk and require the most rigorous clinical trials. Products involving autologous cells, large-scale cultivation, or induced differentiation are deemed medium-risk, permitting confirmatory clinical trials under certain conditions. Autologous cells undergoing only minimal manipulation are classified as low-risk and are subject to the least stringent regulatory requirements. In terms of approval mechanisms, Japan offers a fast-track pathway independent of the standard pharmaceutical approval process. Furthermore, based on preliminary safety and efficacy data, products can obtain conditional or time-limited approval, allowing for continued data collection during clinical use to verify efficacy before achieving full market authorization.

Unlike the United States and the European Union, which typically require confirmatory data from large-scale Phase III clinical trials, South Korea permits “conditional approval” for market entry based on Phase II or even smaller-scale clinical trial data, provided the following conditions are met: the therapy is for a life-threatening or severely disabling disease; its expected efficacy is significantly superior to existing treatments; its benefits outweigh the risks; and the applicant commits to completing confirmatory post-marketing studies. Supported by this policy, Cellgram-AMI® became the world’s first approved mesenchymal stem cell therapy product, followed by the successful market approvals of Cupistem® and Neuronata-R®. This policy accelerates patient access and offers a novel regulatory approach for the global cell therapy landscape.

## 7 Discussion

From a global perspective, countries have made different progress in cell therapy research, focusing on distinct areas. The US and South Korea were early pioneers, conducting related clinical trials around 2000. However, the US only began approving cell therapy products after 2010. Although South Korea had several early product approvals, they were only domestic and did not enter the international market (Iglesias-López et al., 2019; Lee and Lee, 2023). In recent years, the majority of new approvals have come from the U.S., China, and Japan. The U.S. leads globally, and many of its products have since gained approval in the EU, China, and Japan. China’s research projects and marketed products are mainly concentrated in immune cell therapy, especially CAR-T. Currently, China has only one MSC product approved in 2025 in the stem cell field, with other drugs remains under development (Cai et al., 2024). The European Union leads globally in the field of genetically modified autologous hematopoietic stem cells.

Research and development activities for cell therapies are highly concentrated in the United States, China, and Europe, which together account for the vast majority of global clinical trials. Many low- and middle-income countries and regions lag significantly in basic research, clinical trials, and product approvals for cell therapies, resulting in severe geographical inequality in global patient access. This imbalance is reflected not only in research resources and infrastructure but also in disparities in regulatory systems, technological levels, and funding support, exacerbating global health inequity. It is recommended that organizations such as the WHO and the International Society for Cell Therapy establish global or regional cell therapy R&D and translation alliances to support low- and middle-income countries in enhancing their basic research, clinical trial capabilities, and localized production capacity. Approaches such as technology transfer, personnel training, and platform co-development can gradually narrow the global gaps in R&D and accessibility.

Regarding regulatory systems, a unified global regulatory framework for cell therapy products has yet to be established. Major markets such as the United States, the European Union, China, Japan, and South Korea exhibit significant differences in classification standards, review pathways, and data requirements. This leads to challenges for companies conducting international multi-center clinical trials and global market applications, including duplicated efforts, prolonged timelines, and increased costs. Furthermore, limited regulatory capacity and a lack of specialized review experience for cell therapy products in some emerging markets further constrain product accessibility. It is recommended to strengthen dialogue through international organizations such as ICH and WHO to gradually harmonize the classification, technical requirements, and review standards for cell therapy products. Establishing regional regulatory alliances or mutual recognition mechanisms could reduce duplicate reviews and accelerate global product accessibility. Simultaneously, capacity building and technical support for regulatory agencies in emerging markets should be enhanced.

In terms of technological capabilities, although the global scale of cell therapy R&D continues to expand, there is a clear gradient among countries in terms of technological originality, process maturity, and innovation level. Countries are encouraged to increase long-term investment in foundational cell therapy technologies, such as gene editing, and support original research. It is recommended to establish multinational cooperation mechanisms and launch international research programs focused on technological breakthroughs to facilitate knowledge flow and collaborative innovation.

Regarding funding, the development of cell therapy products requires substantial financial investment. National governments can adopt supportive policies or seek international investment support. From the patient’s perspective, cell therapy products are generally prohibitively expensive, particularly autologous CAR-T therapies, where a single treatment often exceeds hundreds of thousands of dollars, far beyond the affordability of most patients. Although countries like China have partially alleviated payment pressures through national medical insurance negotiations, severe treatment inequality persists globally, with patients in low- and middle-income countries having limited access to advanced therapies. In addition to medical insurance negotiations, innovative payment mechanisms such as outcome-based payment models, installment payments, and risk-sharing agreements can be explored to reduce the burden on patients and healthcare systems. Concurrently, optimizing production processes, automation, and scaling up manufacturing are essential to fundamentally reduce production costs.

The development landscape of the global cell therapy industry is the result of the combined effects of regulatory systems, technological innovation, and financial support. Future breakthroughs in the industry depend not only on the innovative research of scholars but also on the regulatory and supportive policies of relevant authorities. Only through deep integration of scientific regulation and technological innovation, backed by robust financial support, can cell therapy transition from a cutting-edge technology to a broadly accessible and equitable medical solution, ultimately benefiting all people.

## Data Availability

Publicly available datasets were analyzed in this study. This data can be found here: https://clinicaltrials.gov/.
